# Durum Wheat (*Triticum durum* L.) Landraces Reveal Potential for the Improvement of Grain Carotenoid Esterification in Breeding Programs

**DOI:** 10.3390/foods10040757

**Published:** 2021-04-02

**Authors:** María Dolores Requena-Ramírez, Dámaso Hornero-Méndez, Cristina Rodríguez-Suárez, Sergio G. Atienza

**Affiliations:** 1 Instituto de Agricultura Sostenible (CSIC), Alameda del Obispo, s/n, E-14004 Córdoba, Spain; mdrequena@ias.csic.es (M.D.R.-R.); crodriguez@ias.csic.es (C.R.-S.); 2 Department of Food Phytochemistry, Instituto de la Grasa (CSIC), Campus Universidad Pablo de Olavide, Edificio 46, Ctra de Utrera, Km 1, E-41013 Sevilla, Spain; hornero@ig.csic.es

**Keywords:** carotenoids, durum wheat, esters, landraces, lutein

## Abstract

Carotenoids are essential in the human diet for their important functions in health. Besides, they are responsible for the yellow pigments desirable for industrial quality in durum wheat. The remarkable carotenoid content of durum wheat endosperm is mostly due to lutein. However, lutein esters have not been previously detected in durum wheat as in other cereals such as common wheat, tritordeum or *Hordeum chilense*. Esterification increases carotenoid stability and allows greater retention and accumulation through the food chain. Therefore, carotenoid esterification is revealed as a new key target in breeding. We characterized the carotenoid profile of 156 accessions of the Spanish durum wheat collection, searching for landraces with esterification ability. Interestingly, four accessions produced lutein monoesters and diesters. Also, traces of lutein monoesters were detected in eleven accessions. The identification of the first durum wheat accessions with esterification ability reported herein is a remarkable advance for carotenoid biofortification. Furthermore, variation for the relative content of zeaxanthin, α-carotene and β-carotene was also observed. This diversity for the β,ε and β,β branches of the carotenogenic pathway also represents a new opportunity for breeding for specific carotenoids in biofortification programs.

## 1. Introduction

Carotenoids are lipophilic pigments synthesized by photosynthetic organisms and some non-photosynthetic ones such as bacteria and fungi. In plants, these compounds are crucial for certain biological processes, including the assembly of photosynthetic pigments–protein complexes, photoprotection in prevention of photo-oxidative damage and regulation of growth and development [1].

In humans, carotenoids cannot be synthesized and thus, they are considered essential nutrients exerting important functions in health. The consumption of carotenoid-rich foods has also been associated with a lower risk of developing certain types of cancer [2]. In particular, lutein and zeaxanthin have been associated with the alleviation of age macular degeneration [3] and they may play a role in neural system development [4]. Furthermore, vitamin A deficiency is one of the main causes of malnutrition in the world [5]. All carotenoids with unsubstituted β-rings have provitamin A activity, therefore β-carotene is the most potent vitamin A precursor since it has two β-rings [6]. This has promoted the development of biofortification programs towards the enhancement of β-carotene content in rice and maize [7,8,9,10].

Carotenoids are also of great significance for the industrial quality of wheat and related species as they are responsible for the yellowish coloration required for high quality pasta and couscous [11,12]. Similarly, they provide the distinctive golden color of tritordeum (*×Tritordeum martini* A. Pujadas) flour and derived products, contributing to the successful commercialization of this new crop [13].

Lutein is the main carotenoid in the endosperm of Triticeae species, including common wheat (*Triticum aestivum* L.) [14], durum wheat (*Triticum durum* L.) [11,12], tritordeum [15], barley (*Hordeum vulgare* L.) and wild barley (*Hordeum chilense* Roem. and Schult.) [16,17]. However, these species differ in their carotenoid content and profile, and especially in their capacity to produce lutein esters in grain. Thus, whereas diploid and hexaploid wheats are able to synthesize lutein esters, tetraploid wheat seems to be unable to produce them [18]. Besides, within-species diversity has also been reported. In a survey of 138 common wheat genotypes, most of them showed lutein esters in their carotenoid profile and only nine were classified as zero-ester genotypes [19]. The occurrence of lutein esters in common wheat has been further documented [20,21,22]. Thus, in a recent study conducted by Paznocht et al. [20] nine out of twelve common wheat genotypes analyzed were able to produce lutein esters, as happens with the bread wheat line DM5685*B12 during post-harvest storage [21] and “Chinese Spring” at harvest [22]. Carotenoid esters have been identified in other Triticeae species, including tritordeum [20,23], cultivated and wild barley [17,20], spelt (*Triticum spelta* L.) and einkorn (*Triticum monococcum* L.) [18].

The high stability of carotenoid esters compared to the non-esterified carotenoids allows better retention in the food chain [21,24,25,26] and thus, esterification is an interesting target for durum wheat breeding. Indeed, efforts are being conducted at present to transfer this trait from common to durum wheat [27]. The recent identification of the gene responsible for lutein esterification in common wheat (*XAT-7D*) should facilitate this task [28]. However, the existence of sources for lutein esterification in diploid and hexaploid wheat suggests that this could also be the case for the durum wheat gene-pool, although this option has not been sufficiently explored so far.

In this context, landraces are considered an attractive breeding option since they are a reservoir of adaptive and quality traits [29]. Spanish durum wheat landraces have previously shown high levels of polymorphism [30], being valuable sources of diversity for quality traits [31,32] and resistance against pests [33]. We hypothesized that diversity for lutein esterification may exist in durum wheat, and thus we investigated the carotenoid content and profile, including lutein esters in a collection of Spanish landraces.

## 2. Materials and Methods

### 2.1. Plant Material and Field Testing

The research material used was a collection of Spanish durum wheat landraces conserved at the National Plant Genetic Resources Centre (CRF-INIA, Alcalá de Henares, Spain) including the core collection developed by Ruiz et al. [30]. It comprised 156 accessions, 9 of them belonging to the *dicoccon* subspecies and the rest (147) to the *turgidum* subspecies (Supplementary File 1). Additional passport data can be obtained from the Spanish Inventory of Plant Genetic Resources Centre [34]. The field trial consisted of non-replicated plots of two rows 1 m long (20 cm between rows and 60 cm between plots) arranged according to an augmented design with four commercial durum wheat varieties (“Amilcar”, “Kofa”, “Monastir” and “Olivadur”) as control checks. Rainfall during the period March–April–May was 68.4, 45.2 and 35.6 mm, respectively. Fertilization was added just before sowing (27 kg N/ha, 69 kg P/ha) and at tillering (82.8 kg N/ha). Samples were harvested at maturity and grains were stored at −80 °C until the extraction and analysis of carotenoids.

### 2.2. Chemical and Reagents

HPLC-grade acetone was supplied by BDH Prolabo (VWR International Eurolab, S.L., Barcelona, Spain). HPLC-grade deionized water was produced with a Milli-Q Advantage A10 system (Merck Millipore, Madrid, Spain). The rest of the reagents were all of analytical grade.

### 2.3. Extraction of Carotenoids

Carotenoid pigments were extracted from durum wheat grains according to the method of Atienza et al. [35] with some modifications [36]. The resulting extract was evaporated under a nitrogen stream, and the residue was dissolved in 0.5 mL of acetone (HPLC grade) and stored at −30 °C. The entire process was carried out under dimmed light in order to prevent carotenoid photo-degradation.

### 2.4. HPLC Analysis of Carotenoids

Analysis of carotenoids was performed by HPLC as detailed in previous works [37,38]. Quantification was carried out using calibration curves prepared with pigment standards. Lutein esters were assessed as free lutein equivalents. Similarly, the concentration of (*Z*)-isomers of lutein was determined by using the calibration curve for (all-*E*)-lutein. Analyses were carried out in duplicate and performed on the same day of the preparation of the extracts. Data were expressed as µg/g fresh weight (µg/g fw).

## 3. Results and Discussion

**Figure 1:**
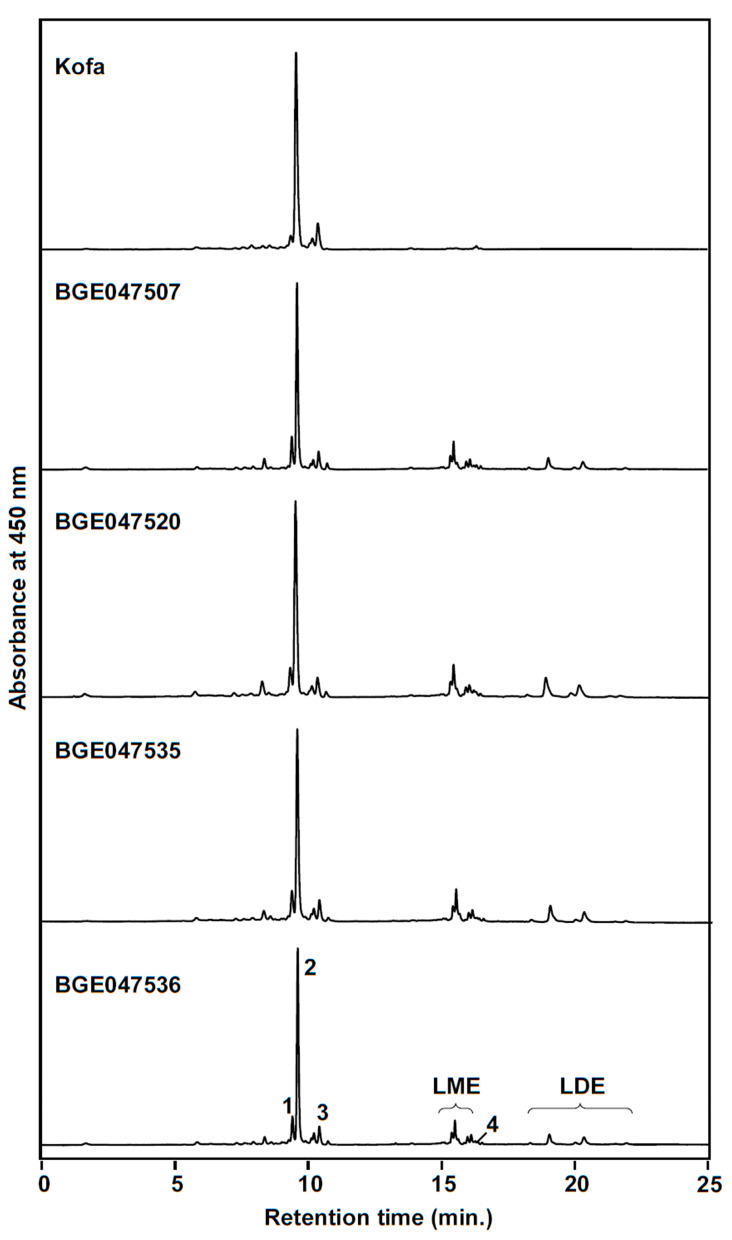
HPLC chromatograms corresponding to the grain carotenoid profile of the durum wheat accessions BGE047507, BGE047520, BGE047535 and BGE047536 presenting carotenoid esters. Cultivar Kofa is included as a zero-ester control. Peak identities: 1, (all-*E*)-zeaxanthin; 2, (all-*E*)-lutein; 3, (*Z*)-lutein isomers ((9-*Z*)-lutein and (13-*Z*)-lutein); 4, (all-*E*)-β-carotene; LME, lutein monoesters; LDE, lutein diesters. Detection wavelength was 450 nm.

The main objective of this work was to explore the carotenoid profile diversity of durum wheat for the identification of landraces with lutein esterification ability. From the 156 genotypes analyzed, most of them were unable to produce significant amounts of lutein esters in agreement with the literature published so far [18,25,36]. Interestingly, four accessions (BGE047507, BGE047520, BGE047535 and BGE047536) produced lutein esters with a significant contribution to the total carotenoid pool, ranging from 26.8% to 31.9% (Table 1). Moreover, both lutein monoesters and diesters were detected in these accessions (Figure 1) and all of them belong to the Spanish durum wheat core collection [30]. 

**Figure 2:**
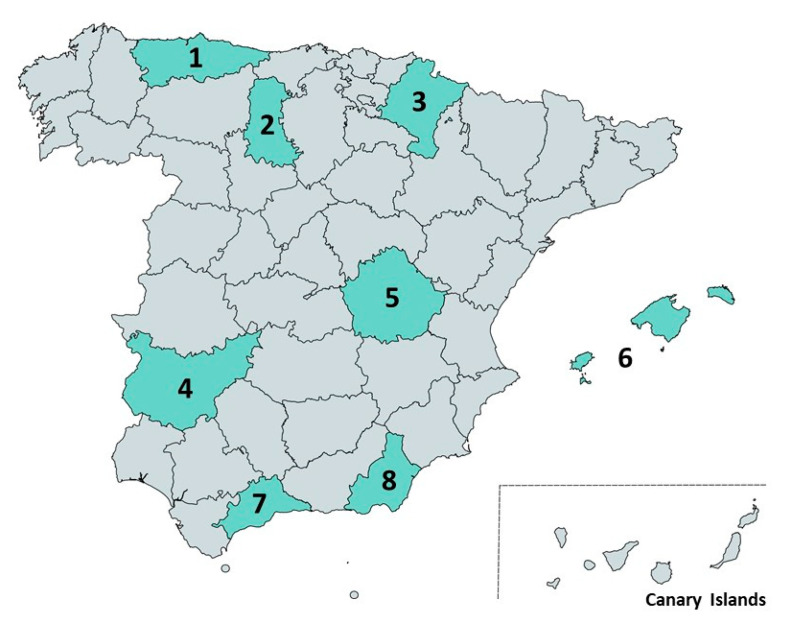
Geographical origin of durum wheat accessions presenting carotenoid esters. Spanish province number: 1. Asturias (BGE045629, BGE047531, BGE048495 and BGE048496); 2. Palencia (BGE047506); 3. Navarra (BGE045645 and BGE047504); 4. Badajoz (BGE047523 and BGE047535); 5. Cuenca (BGE012301 and BGE047524); 6. Islas Baleares (BGE047507 and BGE047536); 7. Málaga (BGE047520); 8. Almería (BGE048494).

Additionally, eleven accessions were identified as cultivars with lower lutein esterifying activity as only traces of monoesters, but not diesters, was detected (Supplementary File 1). Four of these accessions belonged to the *dicoccon* subspecies (BGE012301, BGE045629, BGE045645 and BGE047524), and the rest of them belonged to the *turgidum* subspecies (BGE047504, BGE047506, BGE047523, BGE047531, BGE048494, BGE048495 and BGE048496). No clear association between the geographical origin of the accessions and the presence of carotenoid esters was observed, although two of the accessions producing noticeable levels of lutein esters (BGE047507 and BGE047536) were originally collected in the same production area (Figure 2). A previous study investigated the genetic structure of a durum wheat collection and identified up to seven population groups [39]. As expected, all *dicoccon* accessions belong to the Pop5 group since this population includes accessions of this subspecies. Ten out of eleven accessions (with the exception of BGE047507) belonging to the *turgidum* subspecies were classified in Pop3 group [39] and they correspond to the convariety *turgidum* according to the Spanish Inventory of Plant Genetic Resources [34].

It is noteworthy to highlight that this is the first report of the existence of diversity for carotenoid esterification in durum wheat, since previous studies did not identify any accession producing carotenoid esters (especially diesters) in tetraploid wheats, either durum wheat [18,25,35] or emmer wheat [18]. Therefore, we conducted an additional analysis in the four accessions with high esterified lutein using an independent set of samples cultivated under greenhouse conditions. The results confirmed the production of lutein esters in these accessions (Figure S1).

Esterification is a common mechanism for carotenoid sequestration and accumulation in plants [40,41]. The improvement of the sink capacity and the generation of carotenoid sequestering structures is useful for the enhancement of carotenoid content [42]. Besides, esterified lutein is retained longer than free lutein during post-harvest storage showing higher stability [21,25,43]. Lutein esters are more stable than free lutein during post-harvest storage, but these results have not been confirmed at high temperature regimes (>130 °C) [44]. Nevertheless, considering the differences in carotenoid degradation rates reported in similar experiments, it is clear that new technologically optimized processing approaches are needed, as suggested by Paznocht et al. [44,45]. In this context carotenoid esterification can be considered a new key target for increasing carotenoid retention [26].

The identification of *XAT* genes (*xanthophyll acyl-transferase*) as responsible for lutein esterification in common wheat (*XAT-7D*) [28] and tritordeum (*XAT-7Hch*) [46] has opened new possibilities for the enhancement of carotenoid content in cereals. In fact, current efforts are being conducted for the introgression of *XAT-7D* into durum wheat [27] and a similar approach can be conducted with *XAT-7Hch* since *H. chilense* genes can be transferred to wheat background [47,48,49]. Similarities between *XAT-7D* and *XAT-7Hch* suggest the existence of a common esterification mechanism in the Triticeae species [46], however, preliminary results in our lab provide no evidence of a homoeologue in chromosomes 7A and 7B of durum wheat, so further confirmation is still required. In any case, the landraces identified in this work constitute an excellent source for the improvement of lutein esterification in durum wheat.

The detailed carotenoid profiles are shown in Table S1. Lutein (mainly the (all-*E*) isomer with small amounts of (*Z*)-isomers) was the major carotenoid pigment detected in all samples, accounting for 80%–93% of the total carotenoid content (Table S2). The predominant presence of lutein in durum wheat endosperm is consistent with previous studies (reviewed in Ficco et al. [11], Colasuonno et al. [12] and Rodríguez-Suárez et al. [15]). The durum wheat varieties described above in which the occurrence of lutein esters have been reported for the first time involved majorly palmitic and linoleic acids, which is in agreement with previous reports for other Triticeae species [17,18,49,50].

Durum wheat landraces showed lower total carotenoid contents compared to the commercial varieties used as controls, with a mean of 2.405 µg/g (ranging from 1.032 to 5.274 µg/g), while the commercial varieties presented a mean value of 3.439 µg/g (ranging from 2.178 to 4.391 µg/g). The yellow pigment index, which is routinely used as a selection criterion in durum wheat breeding programs, is due to carotenoids (reviewed in Ficco et al. [11] and Rodríguez-Suárez et al. [15]). As a result of the selective pressure exerted by breeding, modern cultivars obviously show an enhanced carotenoid content compared to older accessions conserved in germplasm banks [51,52].

The carotenoid profile analysis also detected (all-*E*)-zeaxanthin, and minor amounts of (all-*E*)-β-carotene and (all-*E*)-α-carotene. The contribution of zeaxanthin to the total carotenoid pool ranged between 6.27%–18.37% (mean value of 10.88%) (Table S2), which is in agreement with previous studies, and constitutes a conserved trait in durum wheat [35,53]. These results are similar to those found in *H. chilense*, with 12.1% of the total carotenoid pool on average [50,54]. Notably, BGE018675, BGE045643 and BGE045657 clearly showed higher zeaxanthin relative contents (17.72%, 18.37% and 17.36%, respectively) and subsequently lower lutein content than the average (81.42%, 80.48% and 81.83%, respectively). The other three accessions, BGE018321, BGE045628 and BGE045633, stood out for their higher relative β-carotene content (1.55%, 1.51% and 1.78%, respectively). It is also remarkable that α-carotene traces were detected in the endosperm of some durum wheat landraces, a carotenoid that seems to be absent in Tritordeum and *H. chilense* [54,55], being that BGE030921 is the accession with the highest α-carotene content with 0.068 µg/g contributing 2.1% to the carotenoid pool.

**Figure 3:**
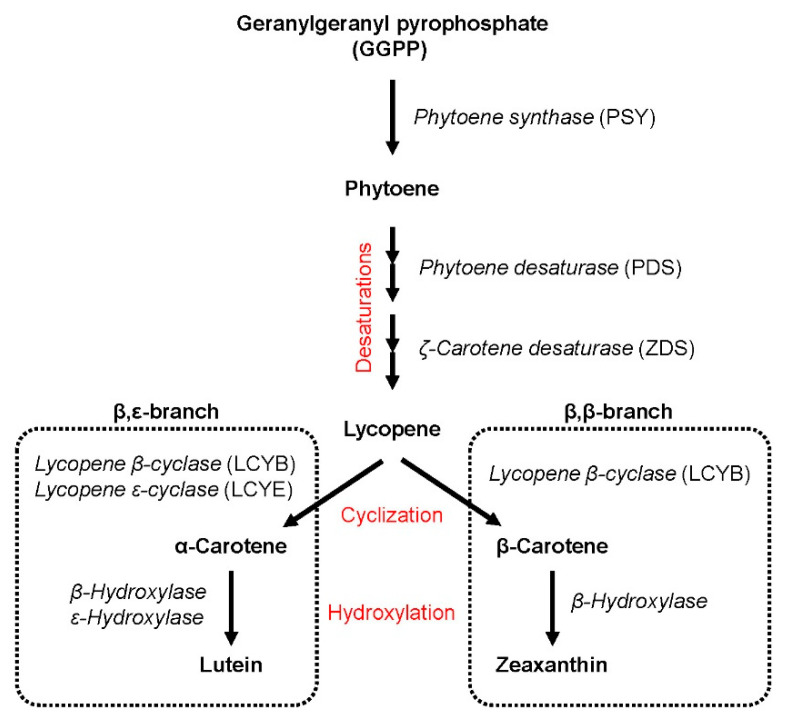
Carotenoid biosynthetic pathway in Triticeae species.

**Figure 4:**
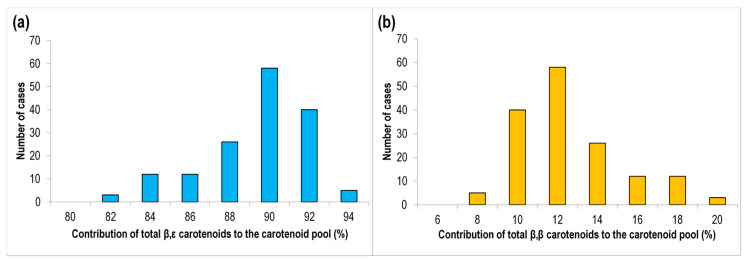
Contribution of β,ε-branch carotenoids (**a**) and β,β-branch carotenoids (**b**) to the total carotenoid pool (%) in the Spanish durum wheat collection.

Lutein and α-carotene are derived from the β,ε-branch of the carotenoid pathway while zeaxanthin and β-carotene are produced from the β,β-branch (Figure 3). Carotenoids from the β,ε-branch accounted for the 88.3% of the total carotenoids in the collection although a continuous variation was observed between the minimum (80.6%) and maximum (93.1%) values (Figure 4a). Accordingly, a similar situation was observed in the β,β-branch (Figure 4b). As described above, accessions with a lower contribution of lutein to the total carotenoid pool present a higher contribution of zeaxanthin, which could be due to an over-activation of the β,β-branch of the biosynthetic pathway and/or a lower degradation after zeaxanthin synthesis [56]. In addition, the low levels of β-carotene in durum wheat suggest that the hydroxylation stages leading to the formation of zeaxanthin are favored, as previously observed in *H. chilense* [54]. The diversion of carotenoids biosynthesis towards each branch is regulated by *lycopene ε-cyclase* (*LCYE*) and *lycopene β-cyclase* (*LCYB*) enzymes [56]. *LCYE* has been associated with differences for lutein content in durum wheat [57] and *LCYB* can be useful for the development of new cultivars with high β-carotene content in wheat [12]. This approach has been successfully applied in maize where natural variation for *LCYE* has been exploited to alter the flux from the β,ε-branch to the β,β-branch [58] in order to develop provitamin A-biofortified maize [7]. Our results reveal the existence of diversity for the relative contribution of carotenoids from each branch to the total carotenoid pool within the collection analyzed. This could be related to the existence of variability at *LCYE* and/or *LCYB* (Table S3) which should be further investigated and could be exploited as successfully applied in maize.

## 4. Final Remarks and Conclusions

Considering the importance of esterification in carotenoid retention throughout the food chain, the efficient utilization of this mechanism constitutes a promising target for carotenoid biofortification in durum wheat. The identification of the first durum wheat accessions with a significant contribution of lutein esters to the total carotenoid profile reported in this work is a remarkable progress in this field (despite the yield potential of these landraces being much lower than commercial varieties). In particular, the four accessions with the ability to produce lutein esters (both monoesters and diesters) can be used as donor sources in durum wheat biofortification programs, being a complementary approach to the ongoing projects trying to transfer *XAT-7D* or *XAT-7Hch*. Additionally, the diversity observed for β,ε and β,β carotenoid biosynthesis branches may be also exploited for targeting specific carotenoids in biofortification.

## Figures and Tables

**Table 1:** Carotenoid composition of durum wheat accessions with lutein esters.

		Lutein Monoesters ^2^		Lutein Diesters ^3^						Contribution to the TC Pool (%)	
Accession	Free Lutein ^1^	Lut ML	Lut MP	Lut DL	Lut PL	Lut DP	Total Lutein ^4^	Rest of Carotenoids ^5^	Total Carotenoids (TC) ^6^	Free Lutein	Esters
BGE012301	1.169	0.013	0.004	-	-	-	1.186	0.233	1.419	82.4	1.2
BGE045629	1.648	0.019	0.006	-	-	-	1.673	0.252	1.925	85.6	1.3
BGE045645	1.795	0.018	0.005	-	-	-	1.818	0.242	2.060	87.1	1.1
BGE047507	0.977	0.180	0.087	0.079	0.065	0.016	1.405	0.164	1.569	62.3	27.2
BGE047504	1.418	0.016	0.005	-	-	-	1.439	0.282	1.721	82.4	1.2
BGE047506	1.502	0.014	0.004	-	-	-	1.520	0.286	1.806	83.2	1.0
BGE047520	1.150	0.205	0.111	0.149	0.127	0.024	1.766	0.167	1.933	59.6	31.8
BGE047523	1.204	0.020	0.005	-	-	-	1.228	0.130	1.358	88.7	1.8
BGE047524	1.759	0.020	0.007	-	-	-	1.786	0.203	1.989	88.4	1.3
BGE047531	2.274	0.019	0.007	-	-	-	2.300	0.289	2.589	87.8	1.0
BGE047535	1.327	0.249	0.123	0.161	0.127	0.028	2.015	0.198	2.213	60.0	31.1
BGE047536	1.480	0.232	0.125	0.121	0.113	0.026	2.097	0.211	2.308	64.1	26.8
BGE048494	2.206	0.031	0.013	-	-	-	2.251	0.254	2.505	88.1	1.8
BGE048495	2.289	0.020	0.004	-	-	-	2.313	0.253	2.566	89.2	0.9
BGE048496	2.198	0.021	0.007	-	-	-	2.227	0.235	2.462	89.3	1.1

^1^ Free Lutein = (all-*E*)-lutein; 3 + (*Z*)-lutein isomers ((9-*Z*)-lutein and (13-*Z*)-lutein). ^2^ Lutein monoesters = Lut ML (Lutein monolinoleate) and Lut MP (Lutein monopalmitate). ^3^ Lutein diesters = Lut DL (Lutein dilinoleate), Lut PL (Lutein linoleate-palmitate) and Lut DP (Lutein dipalmitate). ^4^ Total lutein includes free lutein, lutein monoesters and lutein diesters. ^5^ Rest of carotenoids = (all-*E*)-zeaxanthin + (all-*E*)-α-carotene + (all-*E*)-β-carotene. ^6^ Total carotenoids (TC) = Total lutein + Rest of carotenoids. “-“ = not detected.

## Data Availability

Data is contained within the article.

## Supplementary Materials

The following are available online at https://www.mdpi.com/article/10.3390/foods10040757/s1, Table S1: Detailed carotenoid composition (Absolute values) of the durum wheat collection analyzed; Table S2: Detailed carotenoid composition (Relative values) of the durum wheat collection analyzed; Table S3: *Lycopene β-cyclase* and *lycopene ε-cyclase* genes retrieved from the *Triticum turgidum* and *Triticum dicoccoides* reference genomes (http://plants.ensembl.org/index.html, accessed on 1 April 2021). Figure S1: Validation of accessions producing esterified lutein.
